# Psychological functioning and well‐being before and after bariatric surgery; what is the benefit of being self‐compassionate?

**DOI:** 10.1111/bjhp.12532

**Published:** 2021-05-12

**Authors:** Johanna Eveliina Pyykkö, Ömrüm Aydin, Victor E. A. Gerdes, Yaïr I. Z. Acherman, Albert K. Groen, Arnold W. van de Laar, Max Nieuwdorp, Robbert Sanderman, Mariët Hagedoorn

**Affiliations:** ^1^ Department of Health Psychology Faculty of Medical Sciences University of Groningen University Medical Center Groningen The Netherlands; ^2^ Department of Internal Medicine Spaarne Gasthuis Hoofddorp The Netherlands; ^3^ Department of Vascular Medicine Amsterdam UMC The Netherlands; ^4^ Department of Surgery Spaarne Gasthuis Hoofddorp The Netherlands; ^5^ Department of Experimental Vascular Medicine Amsterdam UMC The Netherlands

**Keywords:** bariatric surgery, obesity, psychology, self‐compassion, weight loss

## Abstract

**Objective:**

To investigate whether patients’ psychological well‐being (depression, quality of life, body image satisfaction) and functioning (self‐efficacy for eating and exercising behaviours and food cravings) improve 12 months after bariatric surgery and whether self‐compassion is associated with better psychological outcomes and lower weight after bariatric surgery.

**Design:**

Longitudinal, prospective observational study.

**Methods:**

Bariatric patients (*n* = 126, 77.8% female, 46.4 ± 10.8 years) completed the Self‐compassion Scale, Center for Epidemiology Studies Depression Scale, Impact of Weight on Quality‐of‐Life questionnaire, Body Image Scale, Weight Efficacy Lifestyle Questionnaire, Spinal Cord Injury Exercise Self‐Efficacy Scale, and G‐Food Craving Questionnaire pre‐operatively and 12 months post‐operatively. A medical professional measured patients’ weight during each assessment. Data were analysed using repeated measures *t*‐tests and multivariate regression analyses with Benjamini–Hochberg correction for multiple testing.

**Results:**

Patients’ BMI, depression, and food cravings decreased significantly after surgery while quality of life, body image satisfaction, and self‐efficacy to exercise improved. Higher self‐compassion was associated with lower post‐operative depression, greater quality of life, higher body image satisfaction, and better self‐efficacy for eating behaviours (*p*‐values <.05) but not with post‐operative BMI, self‐efficacy to exercise, or food cravings.

**Conclusions:**

Even though pre‐operative self‐compassion was not directly associated with a lower 12‐month post‐operative BMI, it had a positive relationship with patients’ post‐operative well‐being and self‐efficacy for controlling eating behaviour. In turn, this could help patients to manage their health long after bariatric surgery. Further work regarding the role of self‐compassion on long‐term health outcomes would be worthwhile.

**Statement of contribution**

***What*** ***is already known on this subject?***

- Self‐compassion demonstrates beneficial associations with eating behaviours and body image as well as weight loss and weight maintenance (among populations without obesity).
- Highly self‐compassionate patients are thought to be better equipped to deal with lapses in their lifestyle (e.g., dieting) by having less critical and harsh reaction towards themselves afterwards and resuming their diet instead.

***What*** ***does this study add?***

- Depression, quality of life, body image satisfaction, food cravings, and self‐efficacy to exercise improved significantly 12 months after surgery while self‐efficacy to control eating behaviours did not change.
- Higher preoperative self‐compassion was associated with lower postoperative depression, greater quality of life, higher body image satisfaction, and better capability to control one’s eating behaviour, but not with postoperative BMI, self‐efficacy to exercise nor food cravings.

## Background

Bariatric surgery is an effective treatment method for severe obesity, leading to an average of 25% loss of patients’ initial weight at 10 years after the surgery (Maciejewski et al., 2016; Sjöström, 2013). In comparison to traditional weight loss treatments, bariatric surgery results in better weight maintenance, more significant improvements in or even complete remissions of obesity‐related comorbidities and greater improvement in the quality of life and psychological health of patients (Colquitt, Pickett, Loveman, & Gk, 2014; Herpertz et al., 2015; Maciejewski et al., 2016; Sjöström, 2013). Despite its effectiveness, a subgroup of patients reach only suboptimal results. Insufficient weight loss or even weight regain can occur as early as 6 months post‐surgery (Courcoulas et al., 2013; King, Hinerman, Belle, Wahed, & Courcoulas, 2018; Sjöström, 2013), with 20–34% of patients reaching suboptimal weight loss 5 years after bariatric surgery (de Hollanda et al., 2015; Hsu et al., 1998).

Long‐term surgical results are determined not only by surgical and medical somatic factors (such as type of operation and number of medical conditions) but also by psychosocial factors such as personality and motivation (Hsu et al., 1998; Nielsen et al., 2020; van Wezenbeek, van Hout, & Nienhuijs, 2016). Greater post‐operative weight loss has previously been associated with younger age, lower initial body mass index (BMI), greater pre‐operative weight loss, lack of severe psychopathological conditions such as depression and anxiety, and lack of personality disorders (Agüera et al., 2015; Kourounis, Kong, Logue, & Gibson, 2020; Livhits et al., 2012). However, previous research findings regarding the predictive value of psychosocial factors remain inconclusive and sometimes are even contradictory due to methodological and conceptual inconsistencies such as using different outcome measures, varying definitions of predictor variables, small sample sizes and diverse lengths of follow‐up (Bastos et al., 2013; Livhits et al., 2012; Maciejewski et al., 2016; van Hout, Verschure, & Van Heck, 2005; van Wezenbeek et al., 2016). Even the commonly used definitions of successful weight loss, such as total weight loss ≥20% or excess weight loss ≥50% (Grover et al., 2019), may have contributed to the weak conclusions, as they are dependent upon the patients’ initial body weight in contrast to absolute measures of weight loss (van de Laar, 2014). Moreover, the field of obesity research has been criticized for the lack of theoretical understanding of the complex underpinnings of obesity as well as the dominating data‐driven approach of previous studies (Markey, August, Bailey, Markey, & Nave, 2016; Taubes, 2013). To understand why some patients seem to benefit less from bariatric surgery than other patients, this study examined the predictive role of self‐compassion on psychological health outcomes and BMI after bariatric surgery.

Successful outcomes of bariatric surgery include also improvement in psychological factors such as depressive symptoms, quality of life, and body image satisfaction (Wimmelmann, Dela, & Mortensen, 2014). The prevalence of depressive symptoms is higher among bariatric surgery candidates and individuals with obesity compared to the general population (van Hout, van Oudheusden, & van Heck, 2004). Similarly, greater weight is associated with poorer health‐related quality of life (Kolotkin & Andersen, 2017). A recent large‐scale cohort study established a robust association between depression, higher BMI, larger perceived body size, and higher body dissatisfaction. Patients with more severe depressive symptoms and higher BMI were also more dissatisfied with their bodies (Paans, Bot, Brouwer, Visser, & Penninx, 2018). Fortunately, depressive symptoms, body image, and quality of life have been shown to ameliorate after bariatric surgery (Kubik et al., 2013; Van Hout, Boekestein, Fortuin, Pelle, & Van Heck, 2006).

Improvement in psychological well‐being is not only an important criterion for successful bariatric surgery but also leads to enhancement in other areas of health and long‐term weight maintenance. The ability to adopt a healthy lifestyle, including regular exercise and a healthy diet, is considered to be paramount for reaching and maintaining satisfactory weight loss and health outcomes after bariatric surgery (dos Rodrigues, de Vasconcelos, & Gomes, 2020; King et al., 2020; Monpellier, Janssen, Antoniou, & Jansen, 2019; Novelli, Fonseca, Gomes, Dutra, & Baiocchi de Carvalho, 2018). Maladaptive eating behaviours such as binge eating, bulimia nervosa, uncontrollable snacking or ‘grazing’, and night eating are common among bariatric surgery patients (Cella et al., 2019; Jumbe, Hamlet, & Meyrick, 2017; Pinto‐Bastos, de Lourdes, Brandão, Machado, & Conceição, 2019; Williams et al., 2017) and associated with poor weight management and unsatisfactory weight loss after bariatric surgery (Conceição et al., 2017; Martin‐Fernandez, Martin‐Fernandez, Marek, Ben‐Porath, & Heinberg, 2021; Pinto‐Bastos et al., 2019). Furthermore, individuals with a higher BMI experience more food cravings than controls with a normal BMI, and these food cravings were more likely to lead to binge eating (Gendall, Joyce, Sullivan, & Bulik, 1998). Self‐efficacy (i.e., one’s belief in own capability), to adhere to dietary and exercise recommendations, could be essential for long‐term weight maintenance as such self‐efficacy is an important predictor of actual eating and exercise behaviours (Bandura, 1982; Linde, Rothman, Baldwin, & Jeffery, 2006).

Recently, mindfulness, mindful eating, and self‐compassion have received increasing attention as potential methods for assisting weight loss. Self‐compassion, being kind and forgiving towards the self, has potential for being an influential factor for weight loss treatment and management. Self‐compassion is a construct based on the premise that suffering, failure, and inadequacy are an inevitable part of the human life and involves three dimensions: self‐kindness, mindfulness, and common humanity. When confronted with difficulties in life, highly self‐compassionate individuals treat themselves with care and kindness, are mindful of their distress, and understand that all humans experience failures (Neff, 2003). In the context of dieting, self‐compassion training has been shown to facilitate weight loss and weight maintenance, possibly by increasing acceptance towards the self when breaking one’s diet and ability to cope with failures (Adams & Leary, 2007; Mantzios & Wilson, 2015b; Mantzios, Wilson, Linnell, & Morris, 2015). In other words, patients high in self‐compassion may be better able to resume their lifestyle regimen and dietary rules, especially after a failure to keep their diet. Further, mindfulness and self‐compassion are negatively associated with numerous eating behaviours such as fat and sugar consumption (Mantzios, Egan, Hussain, Egan, Hussain, Keyte, & Bahia, 2018), grazing (Mantzios, Egan, Bahia, Egan, Bahia, Hussain, & Keyte, 2018), motivations to eat palatable foods (Keyte, Egan, & Mantzios, 2020), and eating disordered behaviour, and it appears to protect against a negative impact of poor body image on quality of life (Duarte, Ferreira, Trindade, & Pinto‐Gouveia, 2015). Self‐compassion is also associated with health‐promoting behaviours (Sirois, Kitner, & Hirsch, 2015), lower psychopathological symptoms (e.g., depression and anxiety), and higher quality of life (Ferreira, Pinto‐Gouveia, & Duarte, 2013; MacBeth & Gumley, 2012; Van Dam, Sheppard, Forsyth, & Earleywine, 2011). A recently published study also found self‐compassion to provide resilience against weight bias and showed that self‐compassion has a negative association between anxiety and depression among pre‐bariatric patients (Braun et al., 2020). Research on the subject has been mostly restricted to cross‐sectional studies and to testing in populations without overweight or obesity (e.g., students, see Braun, Park, & Gorin, 2016 for a review). Due to the various positive relations between self‐compassion and eating behaviours summarized above, self‐compassion could prove to have an influential role in weight loss and management and be a valuable tool for patients trying to lose weight due to its modifiable nature. The field could benefit from research focusing on populations with obesity motivated to lose weight.

To depict a holistic picture of patients’ health and to provide realistic expectations and adaptations after bariatric surgery, a better understanding of psychological factors is needed. This will aid in identifying and understanding psychological characteristics and mechanisms related to surgical success, as well as the development of post‐surgical interventions to enhance adjustment and success. Therefore, the present longitudinal study had two main objectives: first, to assess whether patients’ psychological well‐being (i.e., body image, depression, quality of life) and functioning (i.e., self‐efficacy for eating and exercise, and food cravings) and weight improved from pre‐operative to 12 months after bariatric surgery; second, to investigate whether self‐compassion facilitates the change (i.e., whether the post‐operative outcomes are better for highly self‐compassionate patients).

## Methods

### Study design and participants

The current study is part of a larger, ongoing, prospective research project, namely the BARIA cohort study (Van Olden et al., 2021). The patients are assessed at the hospital before bariatric surgery (T0), and again six, 12, and 24 months after the surgery (T1, T2, and T3, respectively). The pre‐operative and 12‐ and 24‐month post‐operative assessments include online psychological questionnaires. Predominantly, patients underwent a laparoscopic Roux‐en‐Y gastric bypass. However, if a patient had a medical condition or a strong preference justifying a laparoscopic omega‐loop gastric bypass or laparoscopic sleeve gastrectomy surgery, these were performed instead. For the purposes of the present study, data collected from 1 September 2016 until 30 November 2019 from the pre‐operative T0 and 12‐month post‐operative T2 assessments were used. Prior to commencing the study, ethical approval was granted by the local Medical Research Ethics Committee (MREC), approval code: NL55755.018.15. The study was conducted in accordance with the Declaration of Helsinki and the Medical Research Involving Human Subjects Act (WMO).

Participants were recruited from the outpatient clinics of Surgery and Internal medicine at Medical Centre Slotervaart in Amsterdam and Spaarne Gasthuis in Hoofddorp, the Netherlands. A surgeon, internist, dietician, and psychologist evaluated each patient’s eligibility for surgery. To be included in the study, participants had to be on a waiting list and meet the criteria for bariatric surgery. These criteria require the patients to have a BMI over 40 kg/m^2^ or a BMI over 35 kg/m^2^ with an obesity‐related comorbidity, have attempted to lose weight under supervision, and be 18–65 years old. Moreover, the patients must not have lost more than 5% of their weight six months prior to surgery and less than 3% of their weight one month prior to surgery to be included in the study. Participants were excluded from the study if they had (1) a primary lipid disorder, (2) known genetic basis for insulin resistance or glucose intolerance, (3) any medical and psychiatric condition except for obesity‐related diseases, (4) coagulation disorders, (5) uncontrolled hypertension, and (6) renal insufficiency, or they (7) used alcohol excessively (>14 units/week) or (8) were pregnant or breastfeeding. All bariatric surgery patients who met the eligibility criteria and signed the informed consent were included in the study.

**Figure 1:**
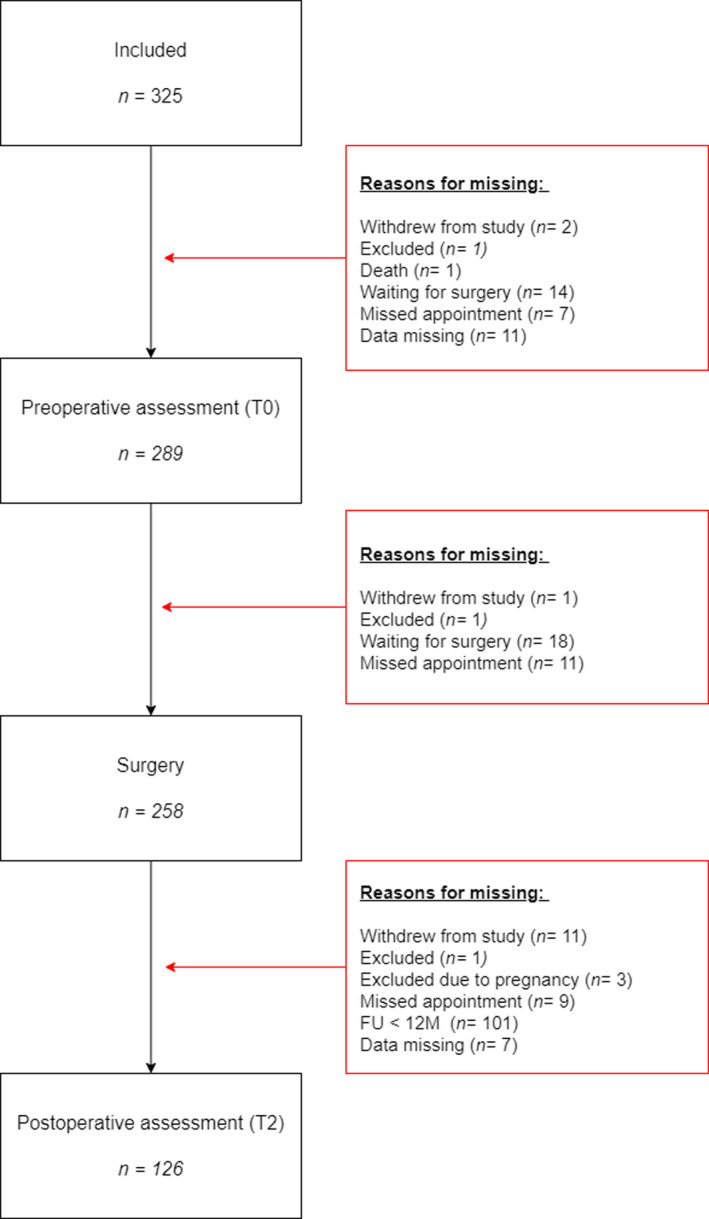
Flowchart of data inclusion.

The data flow chart is presented in Figure 1. Of the 325 patients included in the study, 289 had completed the pre‐operative assessment and 258 had been operated on December 2019. Post‐operative data were missing mainly because patients had not reached their 12‐month follow‐up appointment by the end of data collection (final *n* = 126).

### Measures

During each hospital visit, a medical professional assessed the patients weight, while height was assessed during the pre‐operative assessment. Participants’ socio‐demographic and psychological variables were collected with a self‐report survey including questions about age, sex, educational level, marital status, occupation. and start of obesity, as well as Dutch versions of validated psychological instruments. BMI was calculated as weight in kilograms divided by the square of height in metres (kg/m^2^). Weight loss was measured as change in BMI and percentage of total weight loss (%TWL), calculated as $\%TWL=(\left(Weight_{pre‐op}‐Weight_{post‐op})/Weight_{pre‐op}\right)\times 100$.

#### Self‐compassion

Tendency to be compassionate towards the self when facing difficulties or setbacks was measured with the Self‐compassion Scale Short Form (SCS‐SF; Raes, Pommier, Neff, & Van Gucht, 2011). The scale consists of 12 items such as ‘When something painful happens I try to take a balanced view of the situation’, and responses were given on a 7‐point scale from 1 (almost never) to 7 (almost always). A higher sum score denoted greater feelings of compassion towards oneself. The Cronbach’s alpha for the present study was α = .78 for the pre‐operative scores and α = .84 for the post‐operative score.

#### Depression

Depression was measured with the Center for Epidemiology Studies Depression Scale Revised version (CES‐D; Radloff, 1977), a 20‐item questionnaire for which the responses were given on a 4‐point scale ranging from 0 (rarely or never [less than a day]) to 3 (most or all of the time [5–7 days]). Questions stated for example ‘I was bothered by things that usually don't bother me’ and ‘I enjoyed life’. The total score ranged from 0 to 60 with higher score indicating more severe depression. The internal consistency for the pre‐ and post‐operative scores were α = .83 and α = .91, respectively.

#### Quality of life

Quality of life was assessed with the Impact of Weight on Quality of Life (IWQOL; Kolotkin, Crosby, Kosloski, & Williams, 2001; Kolotkin, Head, Hamilton, & Tse, 1995) questionnaire, which has been developed specifically for populations with overweight. Responses were given on a Likert scale from 1 (never true) to 5 (always true). The scale consists of 31 items such as ‘Because of my weight, I have difficulty getting up from chairs’ and ‘Because of my weight, I experience ridicule, teasing, or unwanted attention’. The total score ranged from 0 to 100 with higher score denoting a better quality of life. The Cronbach’s alphas were α = .92 for the pre‐operative and α = .94 for the post‐operative score in this study.

#### Body image satisfaction

Participant body image satisfaction and perceived attractiveness were measured with the Body Image Scale (BIS; Hopwood, Fletcher, Lee, & Al Ghazal, 2001). The questionnaire consists of 10 items of which the last item referring to surgery scar was omitted from the current analyses because it was not included in the pre‐operative assessment. Responses ranged from 1 (very much) to 4 (not at all) and included items as ‘Do you feel physically attractive?’ and ‘Do you avoid people because of your appearance?’. The total score was coded so that it ranged from 0 to 27 with a higher score indicating greater satisfaction with one’s own body. The Cronbach’s alpha for the present study ranged from α = .81 to α = .73.

#### Self‐efficacy for controlling eating behaviour

Participants’ self‐efficacy for controlling their eating behaviour in specific situations was measured with the Weight Efficacy Lifestyle Questionnaire (WEL‐Q; Clark, Abrams, Niaura, Eaton, & Rossi, 1991). It consists of 20 statements such as ‘I can resist eating even when I am watching TV’, ‘I can resist eating even when I am in pain’, and ‘I can control my eating on the weekends’. An additional answer category was added to ease interpretation to the original 9‐point scale so that the responses ranged from 0 (not confident) to 10 (very confident). Scores were recoded so the total scale ranged from 0 to 100, with 100 meaning the greatest possible self‐efficacy for controlling one’s eating. The Cronbach’s alphas were α = .96 for the pre‐operative and α = .95 for the post‐operative score in the present study.

#### Self‐efficacy for physical activity

Self‐efficacy to be able to perform regular physical activities and exercise was assessed with the 10‐item Spinal Cord Injury Exercise Self‐Efficacy Scale (SCI‐ESES; Kroll, Kehn, Ho, & Groah, 2007). Items start with ‘I am confident…’ and include, for example, ‘…I can find means and ways to be physically active and exercise’ and ‘…that I can be physically active or exercise even when I am tired’. Responses were given on a 4‐point scale from 1 (not at all true) to 4 (always true), and the minimum total score was 10 and maximum 40, higher score indicating better self‐efficacy. Internal consistency was good to excellent with Cronbach’s alphas of α = .89 and α = .94 for the present study.

#### Food craving

The level of craving for food in general was assessed with the G‐Food Craving Questionnaire‐Trait (FCQ‐T, Nijs, Franken, & Muris, 2007). The 21 items are answered on a 6‐point scale from 1 (never or not applicable) to 6 (always) and include statements such as ‘When I eat what I crave, I feel great’ and ‘Once I start eating, I have trouble stopping’. Total score ranged from 0 to 100 with higher score denoting higher desire to eat. The Cronbach’s alphas were α = .95 for the pre‐operative and α = .94 for the post‐operative score in this study.

### Statistical analysis

Data were first analysed with descriptive methods. Change in weight, body image, depression, quality of life, self‐efficacy, and food cravings were tested with repeated measures *t*‐tests. Hierarchical multiple regression analyses (with enter‐method) were used to assess the associations of pre‐operative self‐compassion with post‐surgical outcome variables, controlling for the outcome variable’s pre‐operative value and covariates. Age, sex, pre‐operative BMI, start of obesity (childhood, puberty, and adulthood categories dummy‐coded with adulthood as the reference group), and partnership were included as covariates in the regression analyses when the Pearson correlation between the covariate and outcome variable was significant with *p *< .10. When the assumptions of normality, linearity, and homoscedasticity were not met, a regression analysis with 1,000 bootstrapped samples was used instead. The Benjamini–Hochberg procedure (Benjamini & Hochberg, 1995) with a false discovery rate of .10 was used to correct for multiple testing in the *t*‐tests and regression analyses. In total, 38 tests (*m*) were conducted study wide for the current analyses. Results were deemed significant if the obtained *p‐*value was smaller than the Benjamini–Hochberg critical value. The variance inflation factor was assessed for each predictor to ensure absence of multicollinearity. The statistical software SPSS 25 for Windows (IBM Corp. Released, 2017) was used for all analyses.

## Results

### Descriptive statistics

As shown in Table 1, a majority of patients were female (77.8%) and 46 ± 10.8 years of age on average. Most participants had completed a secondary vocational education (38.9%), were employed (72.2%), and were married or in a registered relationship (52.4%). The laparoscopic Roux‐en‐Y gastric bypass was performed on 120 patients, and the laparoscopic omega‐loop gastric bypass for the remaining six patients included in this study. Participants who were excluded from the current analyses had significantly higher pre‐operative BMI (*Δ* = 0.91 kg/m^2^, *p *= .047) but did not differ in age or sex (*p *> .10).

**Table 1:** Pre‐operative characteristics of the sample (*n* = 126)

	*N*	%
Gender, female	98	77.8
Age (mean, *SD*) [range 18–66 years]	46.4	10.8
Marital status		
Married or registered partnership	66	52.4
Cohabitated	18	14.3
Partner but not living together	9	7.1
Widowed	3	2.4
Separated	8	6.3
Single	22	17.5
Education		
No	1	0.8
General education (high school) *lbo, vbo, mavo, mulo, ulo, havo, vwo, Hbs, gymnasium)*	46	36.5
Secondary vocational education *(mbo, mts)*	49	38.9
Higher professional education (college) *(hbo, hts)*	19	15.1
University	7	5.6
Other	4	3.2
Occupation		
Employed (full or part‐time)	91	72.2
Searching for job	5	4.0
Homework	14	11.1
Voluntary work	3	2.4
Study	2	1.6
Disabled for work	8	6.3
Retired	1	0.8
Other	2	1.6
Obesity Category		
Class I (BMI 30–34.9)	18	14.3
Class II (BMI 35–39.9)	66	52.4
Class III (BMI 40–50)	42	33.3
Start of obesity		
Childhood	39	31
Puberty	25	19.8
Adulthood	54	42.9

### Does patients’ psychological functioning and well‐being change?

As expected, patients’ BMI decreased and all psychological features except self‐efficacy for controlling eating behaviour improved 12 months after bariatric surgery. The mean difference between pre‐ and post‐operative BMI was −11.55 ± 2.81 (*p* < .001), which corresponded to an average total weight loss of 29.5%. Depressive symptoms ameliorated from the pre‐operative mean 8.2 (±6.4) to post‐operative 6.61 (±7.5), whereas food cravings decreased on average by 4.4 (±22.1) units. As for the other psychological outcomes, differences between the pre‐operative and post‐operative values were statistically significant (*p *< .05) for quality of life (30.4 ± 20.9), body image satisfaction (5.5 ± 4.6), and self‐efficacy to exercise (1.7 ± 6.4), as presented in Table 2. The improvements in BMI, quality of life, and body image were large (effect size ≥0.8), and medium for depression, self‐efficacy to exercise, food cravings, and self‐compassion (Cohen, 1988). Only self‐efficacy to control eating behaviour did not change significantly, with the mean difference being 1.2 (±22.4), *p *= .542. Overall, patients’ psychological and physical health improved 12 months after bariatric surgery.

**Table 2:** Weight and psychological functioning before and after bariatric surgery

	T0	T2	Paired differences	*p*	BH	Cohen’s *d*
	*M* (*SD*)	*M* (*SD*)	Mean (*SD*)			
BMI	39.06 (3.46)	27.51 (3.49)	‐11.55 (2.81)***	.000	.003^a^	‐3.33
Depression	8.19 (6.39)	6.61 (7.48)	‐1.58 (6.50)**	.007	.037 ^a^	‐0.23
Quality of life	56.70 (19.96)	87.06 (12.40)	30.36 (20.88)***	.000	.005^a^	1.83
Body image satisfaction	11.93 (4.24)	17.39 (3.38)	5.46 (4.62)***	.000	.008^a^	1.42
Self‐efficacy for controlling eating behaviours	67.83 (19.85)	69.05 (24.48)	1.22 (22.41)	.542	.095	0.05
Self‐efficacy for physical activtiy	32.57 (5.62)	34.28 (6.81)	1.71 (6.35)**	.003	.032^a^	0.27
Food craving	43.30 (20.70)	38.93 (24.64)	‐4.37 (22.09)*	.028	.061^a^	‐0.19
Self‐compassion	59.52 (13.49)	62.13 (12.89)	2.61 (11.07)**	.009	.042^a^	0.20

BH = critical value for the Benjamini–Hochberg correction for multiple testing, (i/m)*Q, where *i* is the rank, *m* is the total number of tests (*m* = 38), and *Q* is the false discovery rate (*Q *= .10).

^a^ Result was still significant after the BH correction (*p* < BH).

*** *p *≤ .001

** *p *≤ .01

* *p *≤ .05.

### Is self‐compassion associated with better outcomes at follow‐up?

The regression analyses showed that self‐compassion had a significant association with lower post‐operative depression (*β *= −.17, *p *= .049), greater quality of life (*β *= .27, *p *= .004), better body image satisfaction (*β *= .25, *p *= .008), and better self‐efficacy for eating behaviour (*β *= .16, *p *= .048) over and above the pre‐operative values of each outcome variable and after the Benjamini–Hochberg correction for multiple testing had been applied. No significant association was found between the pre‐operative self‐compassion and post‐operative BMI (*β *= .08, *p *= .170), self‐efficacy to exercise (*β *= .02, *p *= .787), nor food cravings (*β* = −.11, *p *= .255), after the effect of the selected covariates were controlled for (Table 3). Taken together, these results indicate that patients who were more self‐compassionate before the surgery reached more beneficial outcomes with regard to depression, quality of life, body image satisfaction, and capability to control one’s eating behaviour. Self‐compassion did not account for post‐operative BMI, self‐efficacy to be physically active, and food cravings.

**Table 3:** The results of the hierarchical regression analyses on outcome variables at T2 by self‐compassion and covariates. Only the final models are presented

	*r*	B	95% CI		β	*p*	BH	Sr^2^	*R* ^2^ adj.
Model for T2 BMI^a^									
(Constant)		−2.69	−7.89	2.43		.330	.09		.55
T0 BMI	.67	0.67	0.53	0.81	.66	.001	.00^b^	.42	
Sex	−.30	−1.26	−2.18	−0.20	−.15	.018	.04^b^	.02	
Age	.23	0.08	0.04	0.12	.25	.001	.01^b^	.06	
T0 Self‐compassion	.20	0.02	−0.01	0.05	.08	.170	.07	.01	
Model for T2 Depression^a^									
(Constant)		10.27	−13.85	30.85		.337	.09		.33
T0 CES‐ Depression	.57	0.52	0.30	0.81	.45	.002	.03^b^	.13	
Sex	.17	0.76	−1.04	2.58	.04	.419	.09	.00	
Partnership	−.20	−2.09	−5.32	0.98	−.12	.161	.07	.01	
T0 BMI	−.18	−0.03	−0.40	0.43	−.02	.870	.10	.00	
T0 Self‐compassion	−.40	−0.10	−0.20	−0.01	−.17	.049	.06^b^	.02	
Model for T2 Quality of Life^a^									
(Constant)		67.81	59.61	75.37		.001	.01^b^		.21
T0 QoL	.39	0.15	0.05	0.26	.31	.009	.04^b^	.09	
T0 Self‐compassion	.37	0.19	0.08	0.31	.27	.004	.03^b^	.07	
Model for T2 Body im. sat.									
(Constant)		8.18	1.51	14.84		.017	.04^b^		.18
T0 Body image satisfaction	.31	0.16	0.02	0.30	.21	.025	.05^b^	.04	
Adult vs child start of ob	−.18	−1.50	−2.81	−0.19	−.21	.026	.05^b^	.04	
Adult vs. puberty start of ob	−.03	−0.86	−2.33	0.62	−.10	.253	.08	.01	
T0 BMI	.15	0.11	−0.06	0.28	.11	.203	.08	.01	
T0 Self‐compassion	.36	0.06	0.02	0.11	.25	.008	.03^b^	.05	
Model for T2 SE eating^a^									
Constant		14.19	−5.48	33.99		.172	.07		.27
SE Eating T0	.51	0.56	0.37	0.78	.46	.001	.01^b^	.19	
T0 Self‐compassion	.30	0.28	0.02	0.55	.16	.048	.06^b^	.02	
Model for T2 SE Exercise^a^									
(Constant)		14.49	6.81	21.36		.001	.02^b^		.23
T0 SE exercise	.49	0.59	0.29	0.83	.48	.001	.02^b^	.20	
T0 Self‐compassion	.21	0.01	−0.08	0.11	.02	.787	.10	.00	
Model for T2 Food Craving^a^									
(Constant)		32.92	7.23	58.47		.022	.05^b^		.30
T0 Food craving	.54	0.57	0.36	0.79	.48	.001	.02^b^	.19	
Sex	−.18	−9.19	−18.34	0.24	−.16	.043	.06 ^b^	.02	
T0 Self‐compassion	−.27	−0.20	−0.53	0.14	−.11	.255	.08	.01	

BH = Benjamini–Hochberg critical value, (i/m)*Q, where i is the rank, m is the total number of tests (*m* = 38), and Q is the false discovery rate (*Q *= .10).

^a^ Models with 95% bias corrected and accelerated confidence intervals. CI’s and standard errors based on 1,000 bootstrap samples.

^b^ Result was still significant after the BH correction (*p* < BH).

## Discussion

The present study examined whether weight and psychological well‐being and functioning of patients with obesity changed 12 months after bariatric surgery and whether high self‐compassion facilitated the change. Patients in the current sample lost on average 29.5% of their total weight 12 months after their bariatric surgery, which is in line with previous findings (van de Laar, de Brauw, Bruin, & Acherman, 2016). Men, older patients, and those with higher pre‐surgical BMI had higher post‐operative BMI, which is also consistent with earlier observations (Courcoulas et al., 2018; Marek, Ben‐Porath, van Dulmen, Ashton, & Heinberg, 2017; van Hout et al., 2005). Besides significant weight loss, patients’ psychological well‐being improved on most domains after surgery. Especially changes in BMI, quality of life, and body image showed large effect sizes (Cohen, 1988). Consistent with the literature (Herpertz et al., 2015; Jumbe et al., 2017; Kubik et al., 2013), this research found that patients were overall less depressed, had better quality of life, and were more satisfied with their body image 12 months after bariatric surgery. It has been proposed that post‐operative improvements in psychological health are not attributable to weight loss only but also to patients actively adjusting their lives and changing attitudes and behaviours following bariatric surgery (Jumbe et al., 2017; Kubik et al., 2013). It is possible that not only changes in patients’ attitudes and behaviours but also their motivation are essential components for long‐term surgical success on which further studies could provide more definite evidence.

This study appears to be the first one to examine the role of self‐compassion on health after bariatric surgery. A major finding was that patients who were highly self‐compassionate before their bariatric surgery were less depressed and had greater quality of life, greater body image satisfaction, and higher confidence in their ability to control their eating behaviour 12 months after the surgery. Interestingly, pre‐operative self‐compassion was also a better predictor of post‐operative body image satisfaction than pre‐operative body image satisfaction itself, emphasizing the beneficial effect self‐compassion has on patients’ body image. These findings are consistent with previous studies that have examined self‐compassion among restrictive eaters (i.e., dieters) and eating disordered individuals (e.g., binge eaters). High self‐compassion has been associated with lower eating pathology and weight concerns, higher body appreciation, and intuitive and mindful eating (Braun et al., 2016), as well as healthy eating habits, lower psychopathology, and higher quality of life (Braun et al., 2016; MacBeth & Gumley, 2012; Sirois et al., 2015). Self‐compassion has even been proposed to act as an protective factor against eating pathology and body image disturbances (Braun et al., 2016). Individuals with obesity often have to deal with stigmatization and shame (van Hout et al., 2004). Self‐compassion could help coping with these feelings. For example, self‐compassion has been shown to break the link between shame, body image dissatisfaction, and drive for thinness among women with and without eating disorders (Ferreira et al., 2013). Many of these detrimental factors are also present among dieters and adults who are overweight (Mantzios & Wilson, 2015a). The current study extends these findings to patients with obesity undergoing bariatric surgery and emphasizes the protective role of self‐compassion on psychological well‐being among different populations.

Although the isolation and over‐identification subscales of the self‐compassion scale, but not the total score, have previously been found to be associated with BMI (Mantzios, Egan, Hussain, et al., 2018), the current study did not find a significant association between pre‐operative self‐compassion and post‐operative BMI. During the first post‐operative year, bariatric surgery patients’ weight decreases rapidly due to the surgical alterations of the digestive system (Colquitt et al., 2014). Patients usually reach their lowest weight, on average a loss of 30% of their initial weight, within 2 years after the surgery, after which their weight gradually plateaus within 6–8 years after surgery (de Hollanda et al., 2015; King et al., 2018; Maciejewski et al., 2016; Sjöström, 2013). After the surgical effects have worn off, psychological and behavioural characteristics may become increasingly important for continued weight loss maintenance. Moreover, patients’ psychological characteristics change after bariatric surgery as they are adjusting to their new lifestyle, which could explain why the pre‐operative characteristics themselves do not predict post‐operative weight well. Therefore, longitudinal research is needed to investigate the role of psychological factors throughout the treatment.

Another possible explanations for the insignificant relationship between self‐compassion and BMI is that self‐compassion has an indirect effect on physical health. Highly self‐compassionate patients are better equipped to deal with lapses in their lifestyle but are also better off in terms of their psychological health. For example, it has been found that dieters whose self‐compassion had been increased by a short self‐compassion training were less likely to overeat after breaking their diet. Highly self‐compassionate individuals were less likely to react with criticism and harshness after a failure and therefore experienced less emotional distress after eating high calorie foods (Adams & Leary, 2007; Wasylkiw, MacKinnon, & MacLellan, 2012). They were better able to resume their diet, resulting in better weight outcomes (Adams & Leary, 2007). In the long‐term, these self‐management skills may help to maintain weight loss after bariatric surgery. Taken together, these results suggest that 12 months after bariatric surgery, highly self‐compassionate patients have not only improved more in their psychological well‐being compared to the low self‐compassionate patients, but may also be better able to deal with failure and lead a mindful, balanced lifestyle that could lead to better long‐term health outcomes.

The present findings emphasize the advantages of incorporating interventions targeted at health behaviours for bariatric populations. Self‐compassion can be trained, and enhancing self‐compassion may alleviate eating disordered symptoms and protect and reduce the impact risk factors may have on quality of life and well‐being (Braun et al., 2016). Self‐compassion and mindfulness‐based interventions, such as the Mindfulness‐Based Cognitive Therapy, compassionate mind‐training, and Kg‐Free, lead to an improvement in both self‐compassion as well the targeted health and well‐being outcomes (Ferrari et al., 2019). Similarly, the therapeutic effects have been shown to be significant and effective in improving eating behaviours, eating attitudes, depression, and even weight among adults who are overweight and obese (Rogers, Ferrari, Mosely, Lang, & Brennan, 2017). For example, Acceptance and Commitment Therapy of the Kg‐Free intervention could be especially suitable for bariatric patients as it has been tested among populations with excess weight.

The current study has a few limitations that should be kept in mind when interpreting the results. Even though the measurement tools used to assess psychological characteristics are validated and widely used in research, they are self‐report instruments which could introduce bias to the data. The complete psychological questionnaire used in the current study was lengthy as it included nearly 300 questions and could have led patients becoming fatigued or bored. Therefore, patients were instructed to take breaks while filling in the survey and the medical staff ensured patients took regular breaks while filling in the questionnaire. The Self‐Compassion Scale Short Form used in this study helped to minimize the burden of the lengthy questionnaire on participants, but at the cost of examining more specific research questions. For example, the self‐compassion subscales (e.g., self‐kindness) cannot be reliably constructed from the short form questionnaire (Raes et al., 2011); thus, the influence of the different elements of self‐compassion may have on eating behaviours and psychological and physical well‐being could not be examined. Finally, the follow‐up data were limited to 12 months after surgery. Generally, a longer follow‐up period is recommended to investigate long‐term effects of bariatric surgery on health outcomes. For the current aims, a longer follow‐up data could have clarified the role of self‐compassion on surgical weight outcomes. As the study is ongoing, these results can be replicated with a longer follow‐up data.

Despite the limitations, the current investigation had several strengths. The prospective, longitudinal research design is a valuable strength of the study. Since the presence of psychopathological conditions are regarded as contraindication for bariatric surgery, patients may feel pressured to give answers that portray them in a positive manner to be accepted for the surgery. As all data were collected after the patients had been accepted for bariatric surgery, patients could answer all questions regarding their psychological health and functioning honestly, rendering the data more reliable. Secondly, all data were collected during hospital visits and with medical staff being present for the whole duration of the psychological and biological assessments. Because all assessments were performed during hospital appointments, very little data were missing.

Further work is required to establish both the role of self‐compassion and its subscales on long‐term psychological and physical outcomes after bariatric surgery, and the viability of including a self‐compassion training into the care of patients with obesity. Besides investigating pre‐operative factors, future studies should include longer follow‐up periods and examine how changes in psychological factors after bariatric surgery are related to post‐surgical health and well‐being. The bariatric field could benefit from in‐depth investigations into the needs, obstacles, and successes bariatric surgery patients face throughout their treatment trajectory, which could be obtained through focus‐group interviews involving both patients and medical professionals.

### Conclusion

Highly self‐compassionate patients may benefit more from bariatric surgery in terms of their well‐being and functioning compared to less self‐compassionate patients. Patients with obesity lost a significant amount of weight and their psychological well‐being and functioning improved 12 months after having undergone bariatric surgery. Higher pre‐operative self‐compassion was associated with lower post‐operative depression, greater quality of life, higher body image satisfaction, and better capability to control one’s eating behaviour, but not with post‐operative BMI, self‐efficacy to exercise nor food cravings. Taken together, these results highlight the potential benefit of high self‐compassion on bariatric surgery outcomes and providing self‐compassion training for bariatric surgery candidates.

## Conflicts of interest

All authors declare no conflict of interest.

## Funding

The BARIA study is funded by the Novo Nordiskundation (NNF15OC0016798).

## Author contributions

Johanna Eveliina Pyykkö (Conceptualization; Data curation; Formal analysis; Investigation; Methodology; Project administration; Resources; Validation; Visualization; Writing – original draft; Writing – review & editing) Ömrüm Aydin (Data curation; Investigation; Project administration; Resources; Writing – review & editing) Victor E.A. Gerdes (Conceptualization; Methodology; Resources; Supervision; Writing – review & editing) Yair I. Z. Acherman (Investigation; Resources; Writing – review & editing) Albert K Groen (Conceptualization; Methodology; Writing – review & editing) Arnold W van de Laar (Investigation; Resources; Writing – review & editing) Max Nieuwdorp (Conceptualization; Funding acquisition; Investigation; Resources; Writing – review & editing) Robbert Sanderman (Conceptualization; Funding acquisition; Methodology; Supervision; Writing – original draft; Writing – review & editing) Mariët Hagedoorn (Conceptualization; Funding acquisition; Methodology; Supervision; Writing – original draft; Writing – review & editing).

## Data Availability

Deidentified study data will be shared upon reasonable request by contacting the corresponding author. The full study protocol is published and available to the public (Van Olden et al., 2021).
